# Efficient cell death induction in human glioblastoma cells by photodynamic treatment with Tetrahydroporphyrin-Tetratosylat (THPTS) and ionizing irradiation

**DOI:** 10.18632/oncotarget.20403

**Published:** 2017-08-23

**Authors:** Peter Hambsch, Yury P. Istomin, Dimitri A. Tzerkovsky, Ina Patties, Jochen Neuhaus, Rolf-Dieter Kortmann, Stanislav Schastak, Annegret Glasow

**Affiliations:** ^1^ Department of Radiation Therapy, University of Leipzig, 04103 Leipzig, Germany; ^2^ Department of Ophthalmology, University of Leipzig, 04103 Leipzig, Germany; ^3^ N. N. Alexandrov Republican Scientific and Practical Center of Oncology and Medical Radiology, 223040 Lesnoy, Republic of Belarus; ^4^ Department of Urology, University of Leipzig, 04103 Leipzig, Germany

**Keywords:** photodynamic therapy, glioblastoma, THPTS, combination, ionizing radiation

## Abstract

**Background:**

So far, glioblastomas cannot be cured by standard therapy and have an extremely poor median survival of about 15 months. The photodynamic therapy (PDT) with next generation photosensitizers, reaching a higher therapeutic depth, might offer a new, adjuvant treatment strategy in brain cancer therapy. Here, we investigated the effect of THPTS-PDT combined with ionizing irradiation (IR) on glioblastoma cells *in vitro* and *in vivo*.

**Results:**

THPTS colocalized to mitochondria and was not found in the nucleus. THPTS (2–20 μg/ml)-PDT significantly reduced the proliferation, metabolic activity and clonogenic survival and induced cell death mainly through apoptosis and autophagy. THPTS-PDT combined with IR decreased the clonogenicity significantly compared to single treatments. THPTS (≤ 300 μg/ml) alone showed no dark toxicity. The maximum therapeutic depth of THPTS-PDT in C6 glioblastomas was 13 mm.

**Materials and Methods:**

Three human glioblastoma cell lines (U-87 MG, A-172, DBTRG-05MG) were incubated with THPTS (1–300 μg/ml) 3–24 hours before laser treatment (760 nm, 30 J/cm^2^). THPTS localization and effects on metabolic activity, proliferation, cell death mechanisms and long-term reproductive survival were assessed. IR was conducted on an X-ray unit (0.813 Gy/min). Results were verified *in vivo* on a subcutaneous C6 glioblastoma model in Wistar rats.

**Conclusions:**

This study demonstrated efficient THPTS-PDT in glioblastoma cells, *in vitro* and *in vivo*. The combinatorial effects of THPTS-PDT and IR are of specific clinical interest as enhanced eradication of infiltrating glioblastoma cells in the tumor surrounding tissue might possibly reduce the commonly occurring local relapses.

## INTRODUCTION

Gliomas account for more than 70% of all brain tumors in humans. The *glioblastoma multiforme* (*GBM*) represents the most malignant histological type and occurs with an incidence rate of 4–5/100.000 per year [1]. Despite of multimodal treatment regimen including surgery, radiotherapy (RT) and chemotherapy, progression free and overall survival is still very poor and the median survival time is only 14.6 months [2]. *GBM* cells are highly radio-resistant and often infiltrate the surrounding brain tissue commonly giving rise to local relapses. Over 80–95% of recurrent *GBM*s occur within 2 cm of the resection margin [3, 4]. Photodynamic therapy (PDT) uses a photosensitizer, which generates cytotoxic species (mostly reactive oxygen) in presence of light and oxygen, resulting in cell damage and cell death [5]. The ability of photosensitizers to specifically accumulate in tumor cells, together with the local application of laser light, offers a highly selective antitumor therapy [6]. Application of PDT in cancer is very limited due to the low tissue penetration of laser light. Traditional photosensitizers show maximal absorbance between 628 and 652 nm reaching therapeutic depths of only 5 mm [4]. In clinical trials, adjuvant PDT in brain tumors achieved an initial delay in tumor progression and improvement of median survival but no improvement of overall survival [7–9]. Therefore, photosensitizers are established only as part of the fluorescence image-guided surgical resection (FIGS) [10, 11].

In this study, we use a new photosensitizer, Tetrahydroporphyrin-Tetratosylat (THPTS), with optimal photosensitzer properties [5] for the first time to treat *GBM* cells with PDT. The maximum light absorption of THPTS in the infrared region at 760 nm together with a high extinction coefficient (105,000 M^−1^cm^−1^) allows better tissue penetration thereby inducing a sufficient excitation also in deeper tissue. This has been demonstrated already in C26 colon carcinoma tissue [12]. THPTS has a water solubility of 20 mg/ml, enabling intravenous, intraperitoneal, or interstitial injection. Its skin photosensitivity is low and lasts only about 24 h. THPTS has a 4-fold positive charge, favoring the accumulation in tumor mitochondria [13]. Previous experiments demonstrated that intraperitoneal doses of 20 μg/g body weight were well tolerated in SCID mice [14] and accumulate preferably in the tumor *versus* normal tissue [15]. Because of its chlorine component, THPTS also has a fluorescence emission at 665 nm after selective excitation at 420 nm [12] thus enabling its use in FIGS. FIGS is already established for 5-aminolevulinic acid (5-ALA), where it has been shown to permit a better resectability of *GBM* and consequently an improvement of survival [10, 16]. Recent studies of combined ionizing radiation and PDT showed synergistic effects encouraging further investigations of adjunctive PDT in irradiated tumors [7, 17–20]. PDT could be easily integrated into standard clinical settings: maximum safe surgical resection using photofluorescence can be followed by PDT and RT to remove residual tumor cells [7, 4]. Additionally, PDT can be both applied and repeated at relapse of previously irradiated tumors [3, 8, 9, 21].

Here we evaluated the potential efficiency of THPTS-PDT and its combination with IR to eradicate *GBM* cells. We applied two different experimental models in order to achieve high clinical relevance. To evaluate treatment effects in a human system *in vitro*, we used three different, well established, human glioblastoma cell lines (A-172, U-87 MG, and DBTRG-05MG); *in vivo* investigations were conducted on a C6 glioma Wistar rat model. Long- and short-term reproductive survival, cell death mechanisms and effects on metabolic activity and proliferation, as well as therapeutic depth have been analyzed to provide preclinical data of this novel treatment approach.

## RESULTS

### Preliminary investigations: THPTS stability, localization, incubation time, and light dose

**Figure 1:**
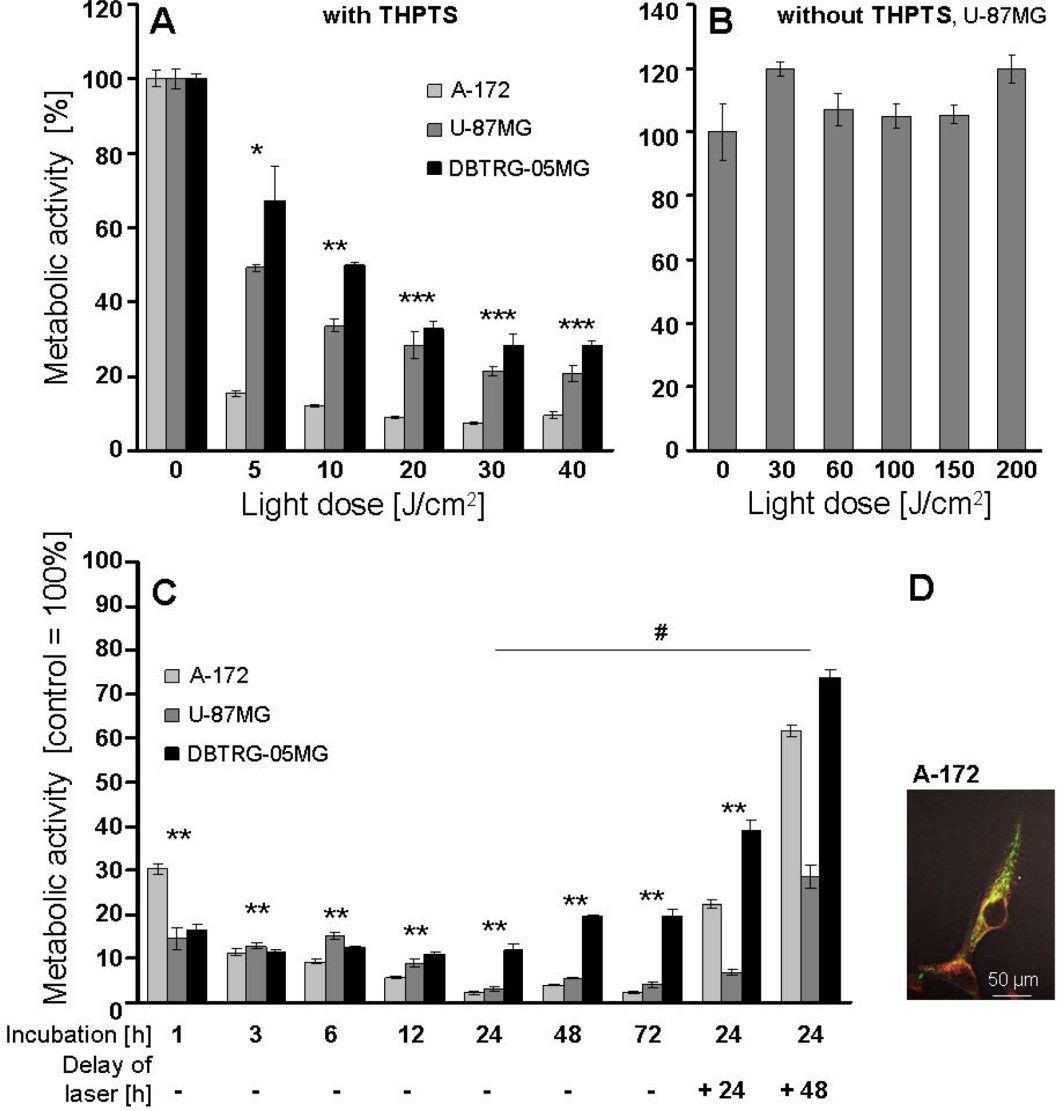
(**A**–**C**) Effects of laser light dose and THPTS incubation time on metabolic activity, measured by WST1 assay. Results show one experiment per cell line, performed in triplicates, mean ± SEM. Joined analysis of three *GBM* cell lines (*n* = 3) is presented. Significant differences compared to control are indicated by asterisks and between groups by hash. (A) The optimal laser light dose was evaluated after incubation with 100 μg/ml THPTS (A-172, U-87MG) or 10 μg/ml THPTS (DBTRG-05MG) for 3 hours. (B) The effect of laser light doses of up to 200 J/cm^2^, delivered at max. 80 mW/cm^2^, without THPTS was analyzed exemplarily in U-87MG cells. (C) To evaluate the optimal THPTS incubation time before laser treatment (drug light interval) *GBM* cells were incubated for 1 to 48 hours with THPTS (100 μg/ml for A-172 and DBTRG-05MG, 200 μg/ml for U-87 MG). THPTS was removed by medium change to avoid self-shielding and immediate or delayed (24/48 h) laser application (30 J/cm^2^) followed. (**D**) Colocalization (yellow) of THPTS (red) and mitotracker Green FM (green). Confocal laser microscopy of THPTS and mitotracker Green FM was performed in A172 cells.

By spectral analysis, aqueous THPTS solutions were found to remain stable for 6 days at 4°C and for 3 weeks if frozen at −20°C (data not shown). Evaluation of the optimal light dose for THPTS-PDT using laser light doses of 5–40 J/cm^2^ delivered at 20–80 mW/cm^2^ showed dose-dependent inhibition of metabolic activities after application of 100 μg/ml THPTS in A-172, U-87 MG and of 10 μg/ml in DBTRG-05MG cells for 3 hours. A significant effect was achieved already at 5 J/cm^2^, *p* ≤ 0.05. Maximal inhibition was seen at 30 J/cm^2^, *p* ≤ 0.001, joint analysis of three cell lines, which was therefore chosen for all subsequent experiments, Figure 1A. Without THPTS, light doses between 30 and 200 J/cm^2^, delivered at a fluency rate of 20–80 mW/cm^2^ had no effect on the metabolic activity compared to untreated controls, tested in the U-87 MG cell line exemplarily, Figure 1B.

To evaluate optimal THPTS incubation and turnover time, A-172, U-87 MG and DBTRG-05MG cells were incubated for 1, 3, 6, 12, 24, 48 and 72 hours with THPTS followed by medium change and immediate laser application (30 J/cm^2^). THPTS incubation intervals of 1 to 72 hours resulted correspondingly in a strong decline of metabolic activity, *p* ≤ 0.01, joint analysis of three cell lines, with most consistent values obtained after THPTS incubation for 3 to 24 h, Figure 1C. If laser application was delayed for + 48 hours after THPTS removal by medium change, effects on metabolic activity were significantly diminished (*p* ≤ 0.05) indicating a THPTS half_life of 24–48 hours in A-172/ DBTRG-05MG cells, and of > 48 hours in U-87 MG cells Figure 1C. Consequently, 3–24 hours incubation intervals and immediate application of laser light doses after medium change were performed in all subsequent experiments. Confocal laser microscopy revealed colocalization (yellow) of THPTS (red) and mitotracker Green FM (green), indicating the uptake of THPTS into mitochondria. THPTS was also located in the cytoplasma, whereas the nuclear uptake was very low, Figure 1D. THPTS was not excitable by ionizing radiation; effects of IR on metabolic activity did not change with/without THPTS if conducted in darkness (data not shown).

### Metabolic activity and cell proliferation

**Figure 2:**
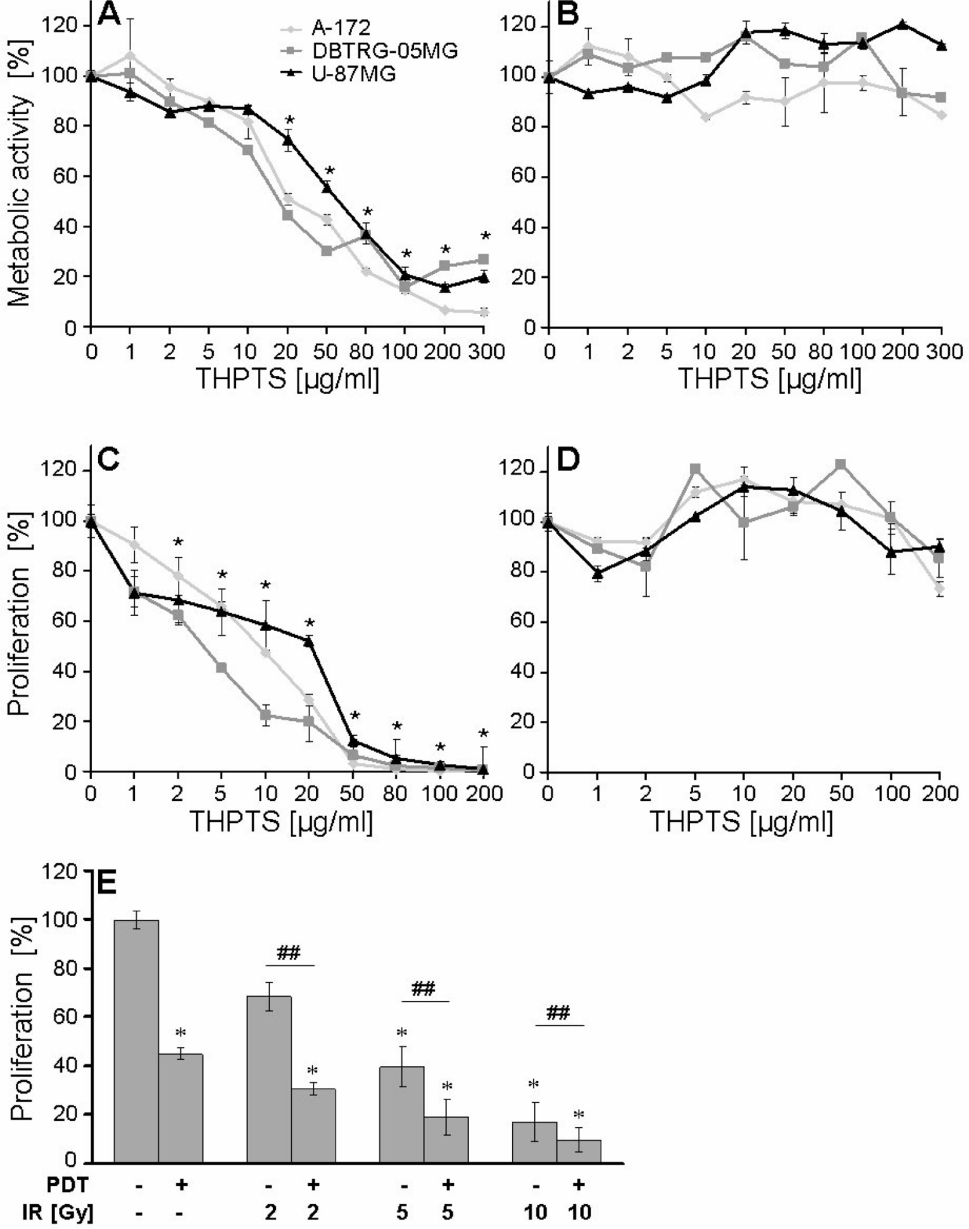
**(A–D)** THPTS concentration-dependent effects of PDT on metabolic activity and proliferation. Results show one experiment per cell line, performed in triplicates, mean ± SEM. Significant differences compared to control are indicated by asterisks and between groups by hash after joined analysis of the three *GBM* cell lines (*n* = 3). Drug light interval was 3 hours. Metabolic activity was measured by WST-1 assay 24 hours after (A) THPTS(1–300 μg/ml)-PDT and (B) after THPTS application without laser light (dark toxicity). (C) Proliferation was measured by BrdU ELISA 48 hours after THPTS (1–200 μg/ml) PDT and D) THPTS application without laser (dark toxicity). (**E**) Combined effect of THPTS-PDT and IR on proliferation. A-172, U-87MG and DBTRG-05MG cells were treated with THPTS-PDT (IC 30) and IR (1 hour after PDT). After 48 h, BrdU ELISA was performed. Results are presented as mean ± SEM, of the three cell lines. Significant differences compared to control are indicated by asterisks. Non-parametric paired Wilcoxon signed-rank test was performed joining all IR dose groups and revealed a significant inhibitory effect of THPTS-PDT + IR *versus* IR alone on cell proliferation; *p* ≤ 0.01, *n* = 3, indicated by hash.

We analyzed the concentration dependency of THPTS-PDT after 24 hours by WST-1 assay using THPTS concentrations from 1–300 μg/ml in A-172, U-87 MG, and DBTRG-05MG cells, Figure 2A.

All three *GBM* cell lines showed a significant inhibition of metabolic activity after application of ≥ 20 μg/ml THPTS, *n* = 3, *p* ≤ 0.05. Concentrations of 100 μg/ml THPTS reduced the metabolic activity by about 70% in all three cell lines. No dark toxicity was observed at THPTS concentrations of ≤ 300 μg/ml, Figure 2B.

The effect of THPTS-PDT on cell proliferation was evaluated by BrdU assay using 0.1–200 μg THPTS/ml. A significant inhibition was found already at ≥ 2 μg/ml, *n* = 3, *p* ≤ 0.05. IC30 doses of THPTS-PDT (concentration leading to 30% inhibition of proliferation) were established (1 μg/ml for A-172, 2 μg/ml for U-87 MG, and 5 μg/ml for DBTRG-05MG, 30 J/cm^2^). A total stop of cell proliferation was achieved at 80 μg/ml THPTS for A-172, 100 μg/ml THPTS for DBTRG-05MG, and 200 μg/ml THPTS for U-87 MG, Figure 2C. No significant inhibition was detected in dark toxicity tests, Figure 2D.

Single treatments (THPTS-PDT) or IR (5 and 10 Gy) as well as combinatorial treatment of THPTS-PDT (IC30) and IR (2–10 Gy) significantly reduced the proliferation compared to untreated control. In addition, non-parametric paired Wilcoxon signed-rank test performed through all IR dose groups (2, 5, 10 Gy), revealed a highly significant inhibitory effect of PDT + IR *versus* IR alone; *p* ≤ 0.01, *n* = 3, Figure 2E. Expansion of time between THPTS-PDT and IR from 1 to 6 hours had no effect (data not shown). IR (5 Gy) decreased the proliferation of DBTRG cells to 54 ± 5% of untreated control level and was not affected by addition of THPTS (IC 30) in the dark (proliferation again at 54 ± 6% of control), meaning that IR alone was not able to induce a photodynamic reaction of THPTS (not illustrated).

### Cell death mechanisms

Repetitive treatment of 7.5 × 10^6^ DBTRG-05MG cells with THPTS (100 μg/ml)-PDT once a day over 3 consecutive days showed 69% vital cells after first treatment by trypan blue exclusion assay. The second application reduced the number of vital cells to 2% and after third THPTS-PDT no vital cells were left (data not illustrated). To define the underlying cell death mechanisms, apoptosis, necrosis, autophagy, and DSB induction were investigated in detail.

**Figure 3:**
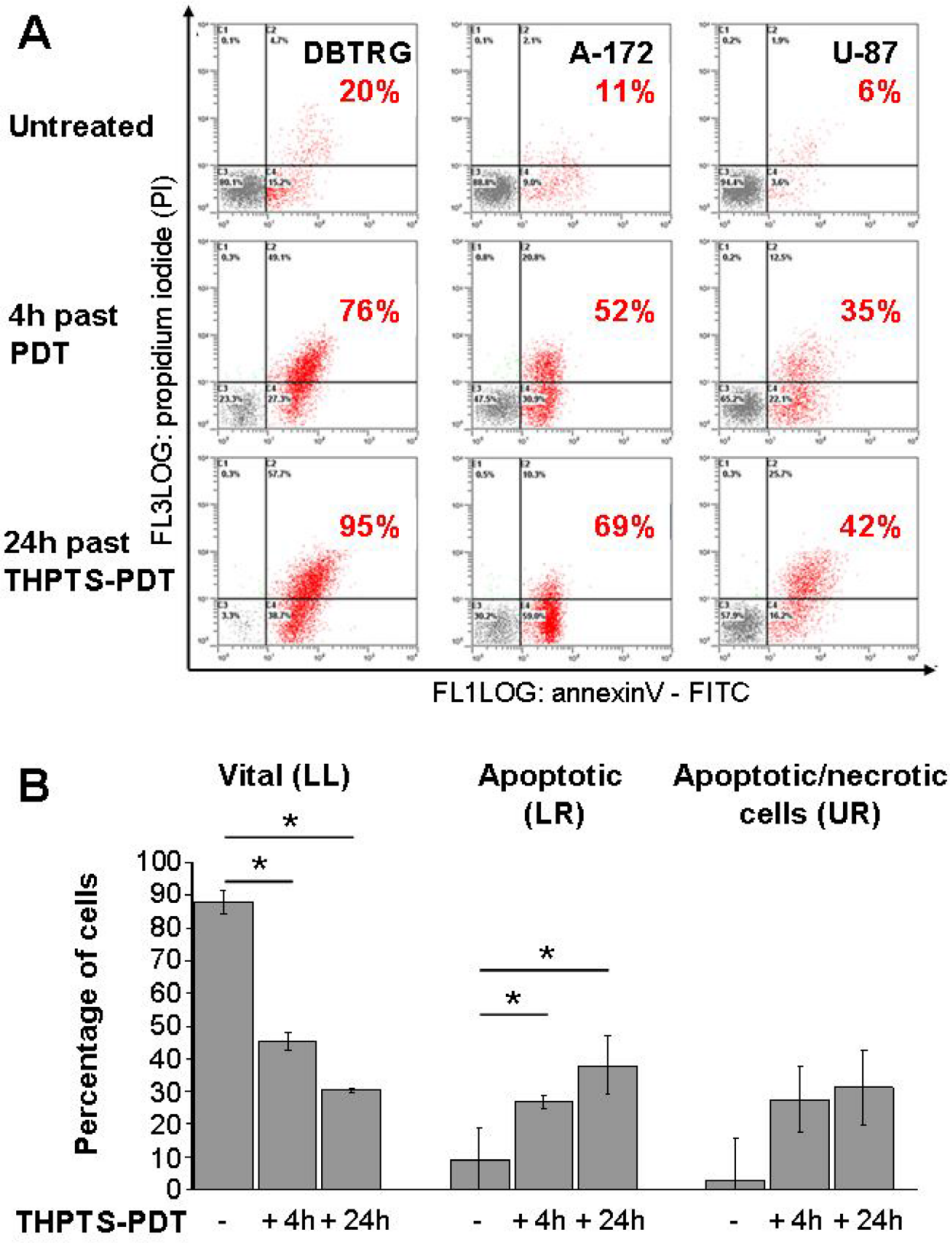
Induction of apoptosis by THPTS-PDT (**A**) Three *GBM* cell lines were stained with FITC Annexin V and PI, 4 and 24 hours after THPTS-PDT (100 μg/ml, 24 hours drug light interval) or laser light application (untreated control). Life cells (Annexin V-/PI-), apoptotic (Annexin V+) cells, apoptotic/necrotic (Annexin V+/PI+) cells and necrotic (Annexin V-/PI+) were quantified by flow cytometry. (**B**) Results of the lower left (LL), lower right (LR) and upper right (UR) part of the dot blots (A) are presented as mean ± SEM of the three cell lines. Joined analysis of the three *GBM* cell lines has been conducted and significant differences compared to control are indicated by asterisks, between groups by hash (*n* = 3).

Flow cytometric evaluation of Annexin/PI-stained cells demonstrated a significant induction of cell death by THPTS(100 μg/ml)-PDT in all three cell lines. Percentage of summarized early apoptotic cells (PI–/ FITC Annexin V+) and late apoptotic/necrotic cells (PI+/FITC Annexin V+) are given for each cell line in red numbers, Figure 3A. The kinetic of the three cell lines is different; DBTRG −05MG cells show the strongest and fastest response to THPTS-PDT. Analysis of pooled data from three cell lines revealed a significant increase of early apoptotic and late apoptotic/necrotic cell death from 12 ± 3% in control cells, to 54 ± 10%, 4 hours and to 69 ± 12%, 24 hours after THPTS-PDT (mean ± SEM, *n* = 3, *p* ≤ 0.05). Cell death is mainly due to apoptosis, as a significant induction of Annexin V+/PI- cells (early apoptosis) 4 hours and 24 hours after THPTS(100μg/ml)-PDT was found (*n* = 3, *p* ≤ 0.05), Figure 3B.

**Figure 4:**
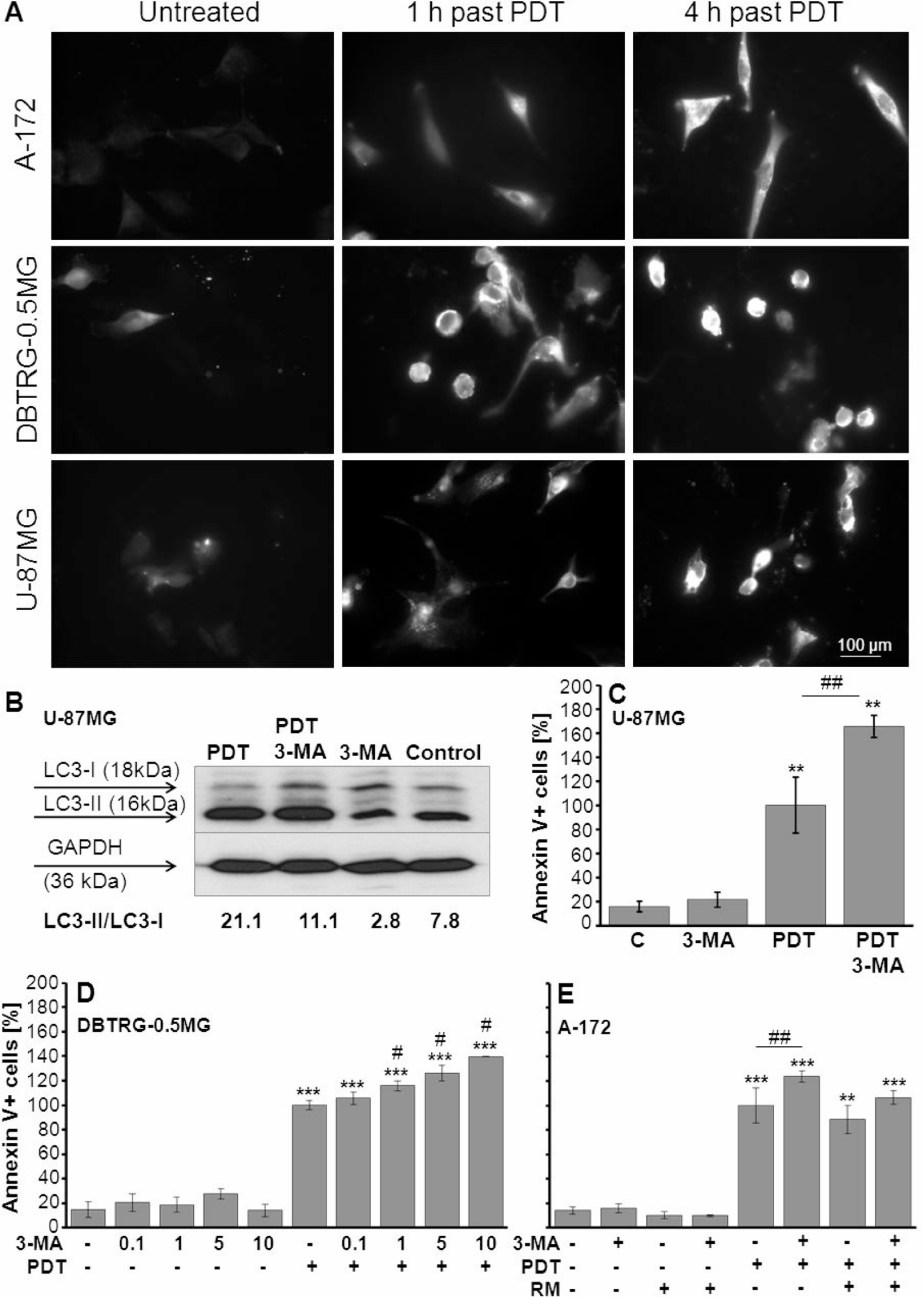
Evaluation of autophagy/apoptosis after THPTS-PDT and application of 3-MA (**A**) Immunofluorescence staining of LC3-II in three *GBM* cell lines 1 and 3 hours after PDT with THPTS (100 μg/ml). (**B**) Western blot analysis of LC3-I/II was exemplarily performed in U87MG cells 24 hours after THPTS-PDT (control = untreated; PDT = THPTS(100 μg/ml)-PDT; 3-MA = application of autophagy inhibitor, 5 mM, 1 h before laser treatment). Protein amounts were normalized on GAPDH levels. (**C**–**E**) Cells were incubated with THPTS (20 μg/ml) for 24 h, then medium was changed and 3-MA (autophagy inhibitor) and/or rapamycin (RM, autophagy inducer) applied 1 hour before PDT. Annexin V and PI staining was executed 24 hours after THPTS-PDT. The relative number (PDT = 100 %) of Annexin V+ (apoptotic and apoptotic/necrotic) cells is presented as mean ± SEM of at least three independent experiments, *n* ≥ 3. Significant differences compared to control are indicated by asterisks, compared to PDT by hash. (C) Enhancement of THPTS-PDT induced apoptosis by 3-MA (5 mM) in U87 MG cells. (D) Dose dependent effect of 3-MA (0.1 10 mM) in DBTRG0.5MG cells. (E) Control, RM abrogates the effect of 3-MA on THPTS-PDT induced apoptosis in A172 cells.

To investigate the effect of THPTS-PDT on autophagy, immunofluorescence staining was applied for the microscopic detection of accumulated LC3-II in autophagolysosomes. Compared to untreated controls, a rise of LC3-II-positive autophagolysosomes was found in all three cell lines 1 hour and even stronger, 3 hours after THPTS-PDT treatment, Figure 4A. To verify these results, western blot analysis was conducted in U-87 MG cells. Indeed, 24 hours after THPTS-PDT treatment a higher ratio of LC3-II to LC3I (21.1) was found compared to untreated control (7.8). Using the autophagy inhibitor 3-MA (5 mM), we were able to reduce the LC3-II/I ratio in the THPTS-PDT treated cells (11.1) as well as in the untreated control group (2.8) confirming the specificity of the western blot analysis, Figure 4B.

To test if the vulnerability of *GBM* cells to THPTS-PDT can be enhanced by inhibition of autophagy, we measured the THPTS-PDT-induced apoptosis using submaximal THPTS concentrations (20 μg/ml) with and without 3-MA. The concentration of 3-MA was optimized using 0.1–10 mM in DBTRG-0.5MG cells, whereby concentrations of ≥ 1 mM 3-MA together with THPTS-PDT reached significant proapototic effects, *n* = 3, Figure 4C. In all three cell lines a significant enhancement of THPTS-PDT-induced apoptosis was found after inhibition of autophagy by 5 mM 3-MA. Thereby, U-87MG cells showed the strongest response with an increase of early apoptotic and late apoptotic/necrotic cells (Annexin V+) by 65.7 ± 9 %, mean ± SEM, *n* = 3, *p* ≤ 0.01%. Also DBTRG-0.5MG and A-172 cells showed significant increases of apoptotic cells, 3-MA + THPTS-PDT-treated cells *versus* THPTS-PDT treated (26 ± 6.5%, *p* ≤ 0.05 and 24 ± 4.7%, *p* ≤ 0.01%, respectively), mean ± SEM, *n* = 3, Figure 4C–4E. To control the specificity of this effect, rapamycin (RM), an autophagy inducer at low concentrations (10–100 nM) and autophagy inhibitor at high concentrations (500 nM), (*In vivo*Gen, manufacturer's protocol) was applied. Rapamycin, 100 nM, was able to abrogate the effect of autophagy inhibitor 3-MA, exemplarily shown in A-172 cells, Figure 4E. Similar response was seen using rapamycin 10 nM, whereas rapamycin 500 nM was found to execute opposite effects (data not shown). The number of necrotic cells (PI+/Annexin V) remained unchanged after application of 3-MA and RM, data not illustrated.

Although nuclear uptake of THPTS was low, nuclear effects cannot be completely excluded. Therefore we analyzed by gamma H2AX-assay if DSB induction is initiated by THPTS(IC30)-PDT. Irradiation (4 Gy) was applied as positive control and resulted in 27.7 ± 1.9 gamma H2AX foci *versus* 4.3 ± 0.48 in untreated control cells after 1 hour, mean ± SEM, *n* = 3 (U-87 MG, DBTRG-05MG, A-172). After 16 h, the majority of foci in the irradiated sample disappeared indicating finalized repair, only 8.5 ± 0.26 foci were counted. No significant induction of gamma H2AX foci/DSB was found in any of the cell lines 1 hour (5.9 ± 0.42 foci) and 16 hours (5.7 ± 0.22 foci) after THPTS-PDT treatment, data not illustrated.

### Clonogenic survival and *in vivo* results

**Figure 5:**
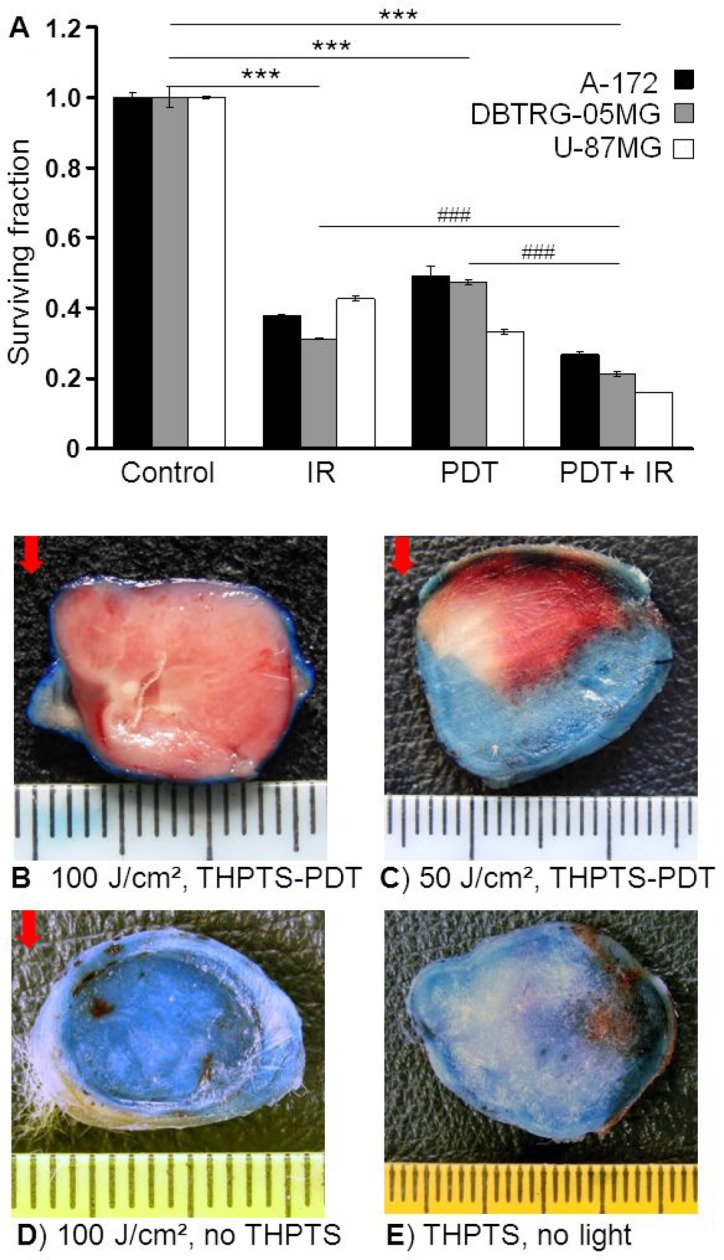
**(A)** Clonogenic survival of human *GBM* cell lines after combined THPTS-PDT and IR. Three *GBM* cell lines were treated with THPTS(IC 50)-PDT (30 J/cm^2^) and/or IR (2 Gy) 1 hour later. Experiments were performed in triplicates for each cell line and joined analysis of all cell lines (*n* = 3) conducted. Significant differences compared to control are indicated by asterisks, between groups by hash. (**B**–**E**) *In vivo* effects of THPTS-PDT (5 mg/kg) in C6-*GBM* tumor-bearing Wistar rats. Evans Blue solution was injected intraperitoneally 24 hours after treatment. Rats were sacrificed 6 hours later and tumors excised. Staining was analyzed in 5 sections of the central part of each tumor. Blue color indicated vital tumor tissue with intact blood supply, whereas dead tissue remains unstained. Red arrow indicates laser light direction. Representative tumors of experimental groups (3 mice/group) are shown. (B) THPTS + photoirradiation 100 J/cm^2^, maximal tumor cell kill was obtained using 100 J/cm^2^ laser light dose resulting in a maximum therapeutic depth of 13 mm. (C) THPTS + photoirradiation 50 J/cm^2^, with a light dose of 50 J/cm^2^ the tumor cell kill was less. (D) Control, application of THPTS without laser light. (E) Control, application of maximum light dose of 100 J/cm^2^ without THPTS.

The three different *GBM* cell lines revealed similar results showing strongest reduction of clonogenic survival in the THPTS(IC50)-PDT+IR(2 Gy) treatment group, mean ± SEM of duplicate experiments. Joined statistical analysis of all three cell lines revealed a highly significant reduction of the surviving fraction by THPTS-PDT (0.43 ± 0.003), THPTS-PDT+IR (0.21 ± 0.002) and by IR (0.37 ± 0.002) alone compared to control (1.00 ± 0.003), *n* = 3, *p* ≤ 0.001. Furthermore, we found the surviving fraction in the combined treatment group, THPTS-PDT+IR significantly reduced compared to single treatments, mean ± SEM, *n* = 3, *p* ≤ 0.001, Figure 5A. To verify the *in vitro* results *in vivo*, we analyzed the vital and dead tumor tissue by Evans blue (EB) staining 24 hours after THPTS-PDT in a C6 *GBM*-Wistar rat tumor model. Six experimental groups (1: THPTS + photoirradiation 100 J/cm^2^, 2: THPTS + photoirradiation 50 J/cm^2^, 3: THPTS without photoirradiation, 4: photoirradiation 100 J/cm^2^, 5: photorirradiation 50 J/cm^2^, 6: control) with 3 mice (*n* = 3)/group were analyzed. Vital tissue was identified by strong EB staining, whereas unstained tumor tissue in the THPTS-PDT treated groups proved effective tumor cell kill. The maximum therapeutic depth of 13 mm (*n* = 3: 12.5, 12.9, 13.0 mm) was reached by a light dose of 100 J/cm^2^. The lower effect reached with 50 J/cm^2^ illustrates clearly, that the therapeutic depth of THPTS-PDT depends on the applied laser light dose, group 1 and 2, Figure 5B, 5C. In controls treated with THPTS but no laser light, group 3 (Figure 5D) or treated with laser light only, 100 J/cm^2^, group 4, (Figure 5E), the strong blue staining identified vital tumor tissue, which was similar in groups 5 and 6, data not illustrated. No adverse effects were observed in any of the animals.

## DISCUSSION

In this study we investigated the efficacy of PDT with the synthetic photosensitizer THPTS to eliminate *GBM* cells *in vitro* and *in vivo.* Furthermore, we combined the THPTS-PDT with the standard postoperative treatment (RT) to investigate the potential of THPTS-PDT as an adjuvant therapy element.

First we optimized the laser light dose, time intervals and THPTS concentration for maximal cell death induction in human *GBM* cells. A relatively low light dose of only 30 J/cm^2^ was found to induce maximal inhibition of metabolic activity in our human *GBM* cell cultures (Figure 1A) and was similarly reported to result in 92% dead cells in human choroidal melanoma (CM) cells [12]. Corresponding to our previous findings [22, 12], no dark toxicity (Figure 2B, 2D) was observed using THPTS concentrations of up to 300 μg/ml. THPTS incubation times between 3 and 24 hours showed similar inhibition of metabolic activity after THPTS-PDT, whereas 1 hour gave lower effects, indicating incomplete THPTS uptake (Figure 1B). These findings are in accordance with our results in RB cells [22] and uptake kinetics in CM cells [12]. The PDT effect on metabolic activity was significantly diminished if laser application was delayed for ≥ 48 hours after removal of THPTS corresponding to the suggested half life of THPTS [22]. If these turnover times are reproduced *in vivo*, an administration of THPTS prior to surgery followed by PDT of the tumor resection cavity, as practiced in 5-aminolevulinic acid PDT [25], seems feasible.

Similar to IR, PDT induces the formation of ROS (reactive oxygen species) [26, 10, 27] potentially leading to DSBs, if produced in the nucleus. We found that in *GBM* cells THPTS is located mainly extranuclear, cytoplasmatic, and intra-mitochondrial after an incubation time of 3 hours (Figure 1D). Accordingly, no DSBs were induced by THPTS-PDT in our experiments indicating that mutagenic side effects are not likely under these conditions. After long incubation periods (24 h) however, some nuclear migration might occur as demonstrated in gall bladder cell lines [14]. The accumulation of THPTS in mitochondria presented here, is favored by the tumor-specific, highly negative mitochondrial membrane potential [28, 27] and has also been shown recently in the Y79 human RB cell line [22], human bladder cancer cells [29], and murine C26 colon carcinoma cells [15].

We demonstrated that THPTS-PDT inhibited the *GBM* cell proliferation significantly already at a concentration of 2 μg/ml (Figure 2C), which is feasible to be reached *in vivo* [14, 15]. Adjuvant PDT and IR might synergize and indeed, successful combination of both therapies (for PDT mostly using indocyanine green or porphyrins) have been reported in different cell lines and also *in vivo* [17–20, 30]. Similarly, here the combination of 2 Gy IR (corresponding to a standard fraction in RT) and THPTS(IC 30)-PDT reached the same effect on *GBM* cell proliferation as a 5 Gy IR treatment (Figure 2E). These effects were independent of the time interval (1–6 h) between THPTS-PDT and IR, indicating that reoxygenation of tumor cells was not of importance. This is in line with recent evidence showing that aqueous THPTS mainly acts via type I electron transfer mechanism involving THPTS reduction rather than oxidation, and producing ROS indirectly through electron donors of the biosystem [31]. THPTS alone, without excitation by light, had no radiosensitizing effect, similarly to the majority of photosensitizers [32].

PDT can induce apoptotic, necrotic and autophagy-associated cell death. Its cytotoxicity largely depends on the type of photosensitizer but also on the cell type [7, 33]. We have already published that THPTS-PDT induces apoptosis in human CM and RB cell lines [15, 22] and could demonstrate here that apoptosis is the major cell death mechanism in human *GBM* cells, too (Figure 3A, 3B). This finding is of high clinical relevance as intracranial necrosis could cause major adverse inflammatory effects in patients. As THPTS is localized in mitochondria, it might induce apoptosis through damage of the mitochondrial membrane by ROS and release of cytochrome c as known for other cationic photosensitizers [5, 7].

THPTS-PDT also led to an accumulation of LC3-II in autophagolysosoms of *GBM* cells indicating the induction of autophagy, which could be partially blocked by 3-MA, an early autophagy inhibitor acting through PI3-kinase inhibition, (Figure 4A, 4B). Beside its role in preventing tumor initiation, autophagy is generally regarded as a strategy of stress evasion and improves (tumor) cell survival in inhospitable circumstances [34, 35]. Recent studies suggest that autophagy inhibitors might sensitize tumor cells to cell death induction [36–38]. Indeed, application of 3-MA enhanced the THPTS-PDT-induced apoptotic cell death in all three *GBM* cell lines (Figure 4C, 4E), indicating that suppression of autophagy might be exploited to further strengthen the PDT effect. Interestingly, repetitive PDT is also an option and was able to kill ≥ log 7.5 of tumor cells, fulfilling an important prerequisite for an effective anti-tumor activity.

The clonogenic assay, considered to be the gold standard of tumor cell response *in vitro*, revealed a 50% reduction of colony forming *GBM* cells by PDT using relatively low THPTS concentrations of 4–10 μg/ml. Similarly to their effect on proliferation, THPTS-PDT combined with IR showed a significant additive reduction of clonogenic survival (Figure 5A). Hence, we assume that THPTS-PDT together with IR might induce lethal damage also in brain tissue infiltrating tumor cells which cannot be eliminated efficiently by surgery or local IR alone. This is of special importance as those cells are the major reason for *GBM* reoccurrence [3, 4].

The efficiency of THPTS-PDT *in vivo* has already been proven in different mouse models including Balb/c mice bearing C26 colon carcinoma [15, 12] and SCID-C.B.17 mice with subcutaneous human bile duct cancer (BDC) [14]. Here we show for the first time that THPTS-PDT is also potent in a *GBM* model using Wistar rats bearing subcutaneous C6 *GBM* tumors (Figure 5B–5E). Additionally we evaluated the therapeutical depth of 13 mm at 100 J/cm^2^ light dose, which corresponds to data reported for THPTS-PDT in a C26 colon carcinoma model [12]. The therapeutic depth rose with increasing light doses and this way might be enhanced even further as known for hematoporphyrin derivatives (HpD) mediated PDT [9].

Here, we report for the first time *in vitro* and *in vivo* data indicating a potential adjuvant application of PDT in brain tumor therapy using the novel photosensitizer THPTS. This infrared-activated photosensitizer kills tumor cells specifically and reaches tissue penetration depths relevant for therapeutic application in brain tumor patients. Of special clinical interest is the enhanced eradication of *GBM* cells by combination of THPTS-PDT with IR.

## MATERIALS AND METHODS

If not otherwise mentioned, experiments have been carried out at room temperature.

### Cell cultures

Human authenticated *GBM* cell lines (A-172, U-87 MG, and DBTRG-05MG) were purchased from and grown as suggested by the American Type Culture Collection (ATCC) for a maximum of 15 passages. Tests to detect mycoplasma were performed in three-month intervals using MycoTrace PCR detection kit (PAA).

### THPTS-PDT *in vitro* and fluorescence imaging

THPTS (C_72_H_70_N_8_O_12_S_4_; molecular weight 1367.77 Da) was purchased from TetraPDT GmbH (Rackwitz, Germany). For laser light delivery, a 3 W diode laser (TetraPDT GmbH) at 760 ± 4 nm was used. After administration of THPTS, cells were handled in the dark except for a filter-armed 600 nm emitting halogen spotlight to prevent excitation of THPTS, according to its spectra [15]. For THPTS-PDT, cells were incubated with and without THPTS for 3 to 24 hours if not otherwise indicated. Before laser treatment, THPTS containing medium was renewed to avoid self-shielding. Light doses of 30 J/cm^2^ were delivered at an intensity of 20–80 mW/cm^2^, if not otherwise indicated. Experimental conditions were kept exactly the same within one specific experiment and its repetitions. The chemical stability of THPTS dissolved in distilled water was evaluated by photometric measurement of its specific absorbance spectrum [12] on an UV-VIS scanning photometer (daily on day 1–6, weekly on week 2 and 3, finally after 2 months). Subcellular localization of THPTS (10 μg/ml) in A-172 cells has been conducted as detailed previously [22].

### Ionizing irradiation (IR)

A 150 kV X-ray machine (DARPAC 150-MC, RayTech) with a dose rate of 0.813 Gy/min was used for IR. In combinatorial treatments IR was conducted 1 hour after PDT if not otherwise indicated.

### Metabolic activity and proliferation

In order to quantify treatment effects on metabolic activity/cell viability by WST-1 assay (Roche) cells were seeded in 96 well plates (5000 to 7000 cells/well) and allowed to adhere for 24 h. THPTS (1–300 μg/ml)PDT, 3 hours drug light interval, was conducted. After 24 h, WST-1 reagent, 10 μl/well, was added and 20 min later absorbance was measured at 450 nm (Tecan Spectra Classic).

Cell proliferation was measured by 5-bromo-2′-deoxyuridine (BrdU) proliferation assay (Roche). *GBM* cells were seeded in 96-well plates (2500 cells/ 200 μl per well) and THPTS(1–200 μg/ml)–PDT, 3 hours drug light interval, was conducted 24 hours later. After 48 hours, medium was renewed, BrdU was added for further 24 hours and BrdU ELISA performed according to manufacturer's instructions. In combinatorial treatments IR was conducted 1 or 6 hours after PDT.

### Apoptosis and necrosis

Apoptotic and necrotic cells were quantified using the FITC Annexin V apoptosis detection kit II (BD Bioscience). Combined FITC Annexin V and propidium iodide (PI) staining was conducted to identify vital cells (PI-/FITC Annexin V-), early apoptotic cells (PI-/ FITC Annexin V+), late apoptotic/necrotic cells (PI+/FITC Annexin V+) and necrotic cells (PI+/ FITC Annexin V-). *GBM* cells in 6-well plates were incubated with THPTS (100 μg/ml) for 24 hours, except for untreated controls. Submaximal THPTS concentrations (20–50 μg/ml) were used to enable the detection of enhanced/reduced effects by autophagy inhibition/autophagy induction. Medium was changed, if applicable autophagy inhibitor 3-methyladenenine, (3-MA, 0.1–10 mM, Merck) and/or autophagy inducer rapamycin (Calbiochem) was added and laser application conducted within 1 hour. Rapamycin is known to induce autophagy via mTOR inhibition at low concentration; concentrations of 10–500 nM were tested according to manufacturer's instructions. After 4 and 24 hours, FITC Annexin V and PI staining were quantified on 5000 cells/sample by flow cytometry (CoulterEpics XL, Beckmann coulter GmbH). Camptothecin, 10 μM, (Sigma) was applied as a positive control for induction of apoptosis. Specificity of antibody binding was controlled by Annexin V block with 15 μg of recombinant Annexin V (BD Bioscience) according to manufacturer's instructions.

### Autophagy

Microtubule-associated protein 1 light chain 3B (LC3) is involved in the autophagic metabolism of cells [23]. LC3-I represents the cytoplasmatic form, which, is processed into LC3-II and recruited to autophagolysosoms and autophagosoms after the induction of autophagy.

For immunofluorescence staining of LC3-II, cells were grown on 8-well chamber slides (7000 cells/ well) and incubated with THPTS (100 μg/ml) for 24 hours. 1 or 3 hours after laser treatment, cells were fixed for 15 min with 2% formaldehyde, washed with PBS, and incubated with bovine serum albumin (BSA), 2% in PBS, for 20 min. Primary mouse anti-LC3 antibody (0231-100/LC3-5F10, NanoTools) or isotype specific control were applied (5 μg/ml) for 1 hour followed by washing and incubation with secondary Cy5-labeled donkey anti-mouse antibody, at 7 μg/ml (Jackson ImmunoResearch) for 1 hour. After washing, nuclei were stained with DAPI (Sigma-Aldrich), 0.5 μg/ml, for 5 min. Sections were embedded in Mowiol 4-88/DABCO (Carl Roth GmbH) and staining was evaluated on a confocal fluorescence microscope (AxioImager Z.1 Carl Zeiss MicroImaging, Tissue Fax software, Tissue Gnostics).

For detection of LC3-I and II by western blot analysis, U-87 MG cells were grown in 75 ml flasks and incubated with THPTS (100 μg/ml). If applicable, 3-MA was added 1 hour before laser treatment. 24 hours later, cells were homogenized for 20 s (Sonoplus Ultrasonic homogenizer HD 2000) in TrisHCL, 50 mM, pH 6.8; 1% SDS; 250 mM sucrose and denatured at 95°C for 10 min. Proteins (20 μg/lane) were separated by SDS polyacrylamide gel electrophoresis, transferred to nitrocellulose membrane and blocked with 5% nonfat milk powder (Carl Roth GmbH) for 60 min. The blot was then incubated with 0.2 μg/ml Mab LC3-2G6 monoclonal mouse antibody (0260-100, NanoTools), recognizing both endogenous LC3 forms LC3-I (18 kDa) and LC3-II (16 kDa), at 4°C overnight and afterwards with 250 ng/ml peroxidase-conjugated horse-anti-mouse IgG (Vector Laboratories) for 2 h. The peroxidase activity was visualized (ECL kit, Amersham Pharmacia) and quantified by gel analyzer software (Media Cybernetics). Membranes were stripped and procedure repeated using a mouse monoclonal glyceraldehyde-3 phosphate dehydrogenase (GAPDH) antibody (Fitzgerald) at 1.25 ng/ml. The LC3-II/LC3-I ratio was calculated after normalization on GAPDH levels.

### Gamma H2AX assay

To detect DNA double strand breaks (DSB), immunfluorescence staining of phosphorylated (Ser139) histone H2AX (gammaH2AX), an early DNA repair protein, was performed as described previously [24]. Therefore, *GBM* cells were seeded in 8-well chamber slides (2000–2500 cells/ well), allowed to adhere for 24 h, and irradiated (4 Gy) or THPTS-PDT treated, except for untreated controls. To allow for repair of potentially induced DSB, submaximal THPTS concentrations (DBTRG-05MG 1 μg/ml, U-87MG 2 μg/ml, A-172 5 μg/ml, 3 hours drug light interval) were used, inhibiting cell proliferation by 30% (IC 30). GammaH2AX staining was executed 1 or 16 hours after laser treatment. A donkey anti-mouse Cy5-labeled secondary antibody (4 μg/ml; Jackson ImmunoResearch) was used here contrary to Patties et al.

### Colony forming assay

Clonogenic assay was performed as previously described [24]. Submaximal THPTS concentrations were used to enable detection of combined effects with irradiation or of repetitive effects. Briefly, cells were treated with THPTS concentrations (10 μg/ml, A-172 and U-87 MG; 4 μg/ml, DBTRG-05MG) corresponding to a 50% inhibition (IC 50) of the plating efficiency (PE), for 3 hours. For laser treatment, living cells (evaluated by trypan blue exclusion test) were transferred to a 6 well plate, lasertreated while in suspension and immediately seeded into 75 cm^2^ cell culture flasks (initially seeded cells). If applicable, IR, 2 Gy, was carried out 1 hour after laser treatment. After 24 hours, vital cells were seeded at two different densities per treatment, in 75 cm^2^ culture flasks, in duplicates. After 14 days, Giemsa-stained colonies of ≥ 50 cells were counted and PE calculated (counted colonies/number of initially seeded cells). The PE of treated cells relative to the PE of the untreated control represents the surviving fraction (SF). For repetitive THPTS-PDT, 7.5 × 10^6^ DBTRG-05MG cells were seeded in 75 cm^2^ cell culture flasks and incubated with 4 μg/ml THPTS for 3 hours. Cells were detached, washed, vital cells counted, laser treated and reseeded. This procedure was repeated after 24 and 48 hours.

### Animal model

Breeding, tumor transplantation and THPTS-PDT treatment of 18 white randomly bred Wistar rats (Rattus norvegicus) were carried out at the N.N. Alexandrov National Cancer Centre (Lesnoy-2, Minsk district, 223040, Republic of Belarus). All manipulations were carried out according to the international and scientific ethical standards detailed in the “Guide on experimental (preclinical) study of new pharmacologic substances”, Health Ministry of Russian Federation, State Pharmacologic Committee of Russian Federation, Moscow, 2000. The animals received a standard diet, had free access to water and were housed at natural 12 hour dark/light cycle. Humidity, illumination and temperature met standard requirements of the in-house sanitary code. C6 experimental rat *GBM* was obtained from the tumor strain collection of the N.N. Blokhin Cancer Research Centre of the Russian Academy of Medical Sciences (Moscow, Russia) and was passed by serial transplantation. Animals were anesthetized by intramusuclar (i.m.) injection of droperidol (5 mg/kg) and fentanyl (0.05 mg/kg) and 10% tumor cell suspension (10% in 0.5 ml Hanks’ solution) was injected subcutaneously into the inguinal region. PDT experiments were carried out, when allograft tumors reached 1–1.5 cm in diameter (6 - 7 days after transplantation).

### THPTS-PDT *in vivo* – C6 GBM in wistar rats

Treatment was performed in 6 experimental groups (1: THPTS + photoirradiation 100 J/cm^2^, 2: THPTS + photoirradiation 50 J/cm^2^, 3: THPTS without photoirradiation, 4: photoirradiation 100 J/cm^2^, 5: photorirradiation 50 J/cm^2^, 6: untreated control) with 3 mice (*n* = 3)/group. THPTS was injected intraperitoneally at a dose of 5 mg/kg, 1 hour before laser treatment. For laser and sham treatments, animals were anesthetized by i.m. injection of droperidol (5 mg/kg) and fentanyl (0.05 mg/kg). Animals were covered with aluminium foil, exposing only the tumor area. Laser treatment was performed using a diode laser (LAMI-Helios; TetraPDT GmbH, Rackwitz, Germany) at 760 ± 5 nm, light dose of 50 J/cm^2^ and 100 J/cm^2^, at an intensity of 150 mW/cm^2^, measured with a Model IL 1400A radiometer/photometer (International Light Inc., Newburyport, MA). After 24 hours, 0.4 ml 1% Evans blue (EB) solution was injected intraperitoneally and 6 hours later, animals were sacrificed. Tumors were excised, and 5 thin slices of the central part of the tumor were cut and examined macroscopically using a morphometrical system (video camera OLYMPUS DP 50, PC Image-Pro program). The unstained area was attributed to dead tissue, whereas the stained area showed tissue with preserved blood supply (vital tissue) as previously described [12].

### Statistical analysis

Statistical analysis was performed by Friedman test (nonparametric repeated measures) or by Kruskal-Wallis one way ANOVA on ranks and posthoc Student-Newman-Keuls test if not otherwise noted. Differences were considered significant at *p* ≤ 0.05 (*), very significant at *p* ≤ 0.01 (**) and highly significant at *p* ≤ 0.001 (***) using SigmaPlot 11.0 software. All data were presented as mean ± standard error of the mean (SEM), n represents the number cell lines or animals.

## Abbreviations

THPTS: Tetrahydroporphyrin-Tetratosylat
PDT: photodynamic therapy
IR: ionizing radiation
RT: radiotherapy
FIGS: fluorescence image guided surgical resection
GBM: *glioblastoma multiforme*
5-ALA: 5-aminolevulinic acid
LC3: microtubule-associated protein1 light chain
DSB: double strand brakes
SF: surviving fraction
HpD: hematoporphyrin derivatives
